# Effect of clove (*Syzygium aromaticum*) and seaweed (*Kappaphycus alvarezii*) water extracts pretreatment on lipid oxidation in sun‐dried sardines (*Rastrineobola argentea*) from Lake Victoria, Tanzania

**DOI:** 10.1002/fsn3.975

**Published:** 2019-02-27

**Authors:** Davis Chaula, Henry Laswai, Bernard Chove, Anders Dalsgaard, Robinson Mdegela, Charlotte Jacobsen, Grethe Hyldig

**Affiliations:** ^1^ Department of Food Technology, Nutrition and Consumer Sciences Sokoine University of Agriculture Morogoro Tanzania; ^2^ Department of Veterinary and Animal Sciences University of Copenhagen Copenhagen Denmark; ^3^ Department of Veterinary Medicine and Public Health Sokoine University of Agriculture Morogoro Tanzania; ^4^ National Food Institute Technical University of Denmark Lyngby Denmark

**Keywords:** antioxidants, clove extracts, *Kappaphycus alvarezii*, Lake Victoria, lipid oxidation, omega‐3 fatty acids, *Rastrineobola argentea*, red seaweed, volatile compounds

## Abstract

Small indigenous fish species play a significant role in food and nutritional security of poor communities in developing countries. Sardines (*Rastrineobola argentea*) are fish species of Lake Victoria known to be a good source of health‐promoting omega‐3 fatty acids. Open sun drying is a common and traditional sardine processing and preservation method. Sun‐dried products suffer from characteristic off‐flavor due to lipid oxidation which discourage product consumption and limit diversification. This study investigated the use of clove (*Syzygium aromaticum*) and seaweed (*Kappaphycus alvarezii)* water extracts as natural antioxidants to impede lipid oxidation in sun‐dried sardines. Lipid oxidation was assessed by peroxide value, volatile secondary oxidation products, and fatty acid profiles. The antioxidant capacity of extracts was evaluated by total phenolic content, 1, 1‐diphenyl‐2‐picrylhydrazyl (DPPH) radical scavenging, and iron (Fe^2+^) chelating ability. Results showed that 5, 10, and 20 g/L clove extracts significantly reduced peroxidation in sun‐dried sardines by 38.7%, 54.6%, and 56%, respectively. Clove extracts resulted in higher retention of omega‐3 fatty acids and lower concentrations of secondary lipid oxidation products as opposed to seaweed counterpart. This research has demonstrated feasibility of pretreating whole, omega‐3‐rich small sardines with natural antioxidants to avert lipid oxidation during sun drying.

## 1 INTRODUCTION

Demands for fish and other aquatic products are increasing because they represent quality proteins and contain health‐promoting long‐chain polyunsaturated fatty acids (PUFAs). Freshwater fish in particular fatty fish species attract great attention as they are known to be a good source of omega‐3 PUFAs namely: docosahexaenoic acid (DHA, C22:6n‐3), eicosapentaenoic acid (EPA; 20:5n‐3), and docosapentaenoic acid (DPA, C22:5n‐3) claimed to have an array of health benefits to humans (Collett, Davidson, Fan, Lupton, & Chapkin, 2001; Finley & Shahidi, 2001; Horrocks & Yeo, 1999; Minihane, Givens, & Gibbs, 2008; Sidhu, 2003; Terry, Lichtenstein, Feychting, Ahlbom, & Wolk, 2001). However, there are considerable challenges for protecting PUFAs during handling, processing, storage, and marketing of fish products. This is due to PUFAs' susceptibility to oxidation that will lead to formation of degradation products, off‐flavors, and other sensory alterations, which in turn reduce acceptance of the products by consumers (Walker, Decker, & Mc Clements, 2015).

Sardines (*Rastrieobola argentea*), popularly known as “*dagaa*” in Tanzania, are tiny, silvery, and fatty freshwater fish species of commercial importance of Lake Victoria providing 72.3% of the total landings by weight (URT, 2015).Traditionally, *dagaa *are preserved by open sun drying and are eaten whole due to their small size. Their proximate composition varies due to environmental factors including the change of seasons and the resultant change of food supply in Lake Victoria (Abdulkarim, Wathondi, & Benno, 2016; Kirema‐Mukasa, 2012). Robert, Mfilinge, Limbu, and Mwita (2014) found that *dagaa *is richer in omega‐3 PUFAs than other species (*Oreochromis niloticus*, *Tillapia zilliii *and *Lates niloticus) *of Lake Victoria. Fresh *dagaa *are reported to contain 13.5%–21.2% omega‐3 PUFAs and 12.5%–22.2% omega‐6 PUFAs (Masa et al., 2011; Mwanja, David, Samuel, Jonathan, & Wilson, 2010). Food products containing omega‐6 and omega‐3 fatty acids are prone to oxidation (Pacheco & Regitano‐D'Arce, 2009; Secci et al., 2016). Particularly, omega‐3 fatty acids have a high number of double bonds and bisallylic carbons with low activation energy favoring hydrogen loss, free radical and hydroperoxides formation (Shahidi & Zhong, 2010).

The traditional low‐cost, low‐technology and weather‐dependent open sun drying of *dagaa* has significant effect on the composition and hence quality of the dried product. Owaga, Onyango, and Njoroge (2010) reported a significant decrease in total fat content (from 14.8% to 13.9%) of *dagaa* after sun drying. This decline of fat content during the sun drying process and subsequent storage may result from rapid lipid oxidation (Huss, 1995; Owaga et al., 2010). Lipid oxidation has detrimental effect on nutritional value and sensory attributes of fish and other products containing omega‐3 fatty acids. Since omega‐3 fatty acids are nutritionally valued, but chemically unstable, researchers seek to understand lipid oxidation and ways to minimize it in omega‐3‐rich products.

Synthetic antioxidants, like butylated hydroxytoluene (BHT), *tert‐*butylhydroquinone (TBHQ), and butylated hydroxyanisole (BHA), are commercially available and widely used to hinder lipid oxidation in food systems. However, there are studies questioning their application in foods due to their potential carcinogenic effects and toxicity (Branen, 1975; Lindenschmidt, Tryka, Goad, & Witschi, 1986; Zheng & Wang, 2001). Because of safety issues and increased consumer interest in natural products, there are up‐and‐coming interests in replacing synthetic antioxidants with ones of natural origin.

Clove and other natural spices, seaweeds, and herbs have the potential to reduce lipid oxidation because they contain anti‐oxidative compounds (polyphenols among others) that may exert anti‐oxidative effect by different mechanisms such as scavenging of free radicals, singlet oxygen quenching, oxygen scavenging, metal chelation, and inhibition of oxidizing enzymes (Dudonné, Vitrac, Coutière, Woillez, & Mérillon, 2009; Shobana & Akhilender, 2000). Attempts have been made to screen different seaweeds for antioxidant activity and phenolic compounds, and tests show that some species are effective in preventing oxidation in real food systems (Farvin & Jacobsen, 2013; Hermund et al., 2015).

Previous studies showed that sun drying of sardines promoted lipid oxidation and its associated undesired flavors and odors, which in turn discourage consumers and limit *dagaa* product diversification. Chemical indicators of lipid oxidation showed that the reactions were pronounced in sun‐dried *dagaa* with production of volatile secondary oxidation products beyond acceptable levels (Chaula et al., 2019). Furthermore, changes in lipid contents with significant decrease in omega‐3 fatty acids in sun‐dried *dagaa* during storage at ambient temperature indicated progression of lipid oxidation (D. Chaula, C. Jacobsen, H. Laswai, B. Chove, A. Dalsgaard, R. Mdegela, & G. Hyldig, unpublished results). The aim of the current study was to evaluate the use of locally available clove (*Szygium aromaticum*) and seaweed (*Kappaphycus alvarezii*) water extracts as a low‐cost pretreatment prior to sun drying of *dagaa* to retard oxidation of omega‐3 fatty acids during sun drying. Knowledge obtained will form the basis for *dagaa *product diversification through incorporation of sun‐dried *dagaa* into other products formulation to enhance nutritional value without offensive off‐flavor resulting from lipid oxidation.

## 2 MATERIALS AND METHODS

### 2.1 Materials

Fresh whole *dagaa *(20 Kg) were collected directly from fishermen at Kijiweni landing site at the shore of Lake Victoria, Tanzania. The *dagaa* were placed in ice in insulated boxes and immediately transported to the National Fish Quality Control Laboratory, Nyegezi, Mwanza for experiment. Dry clove *(Szygium aromaticum*) buds and red seaweed (*Kappaphycus alvarezii*) were obtained from a local market in Zanzibar, transported at ambient temperature to Mwanza, and kept at 5–10°C in a refrigerator.

#### 2.1.1 Preparation of clove and seaweed extracts

For water extraction, 5, 10, and 20 g grounded powder (to pass through a 250 µm sieve) of clove buds and seaweed were mixed with 1 L boiling water with continuous stirring to make 5, 10, and 20 g/L concentrations of clove and seaweed extracts. The mixtures were boiled for 15 min and subsequently cooled to 0–5°C in a refrigerator thereafter gravity filtered to remove the particles present.

#### 2.1.2 Preparation of sun‐dried *dagaa*

For each concentration of clove or seaweed extract, 1 kg of *dagaa *(wet basis) was blanched in boiling water for 10 s. Blanched *dagaa* were soaked in cooled clove or seaweed extracts (1:1 *w*/*w*) for 40 min at room temperature. After that, the fish were removed from the extracts, spread on wire mesh, and sun‐dried on raised platform as it is done by the local fish processors. *Dagaa* samples without clove and seaweed pretreatment were prepared in similar way and used as control. Each treatment experiment consisted of four replicates. For each treatment experiment, 100 g portion of whole fish was made into mince using a mixer (Moulinex Moulinette S type 643 02 210, Hamburg, Germany). The fish mince was then stored at −40°C awaiting analysis.

### 2.2 Methods

#### 2.2.1 Dry matter content and lipid extraction

The dry matter content for fish samples, clove, and seaweed powders was determined by weighing after drying a sample of approximately 2 g of homogeneous fish mince and powder at 105°C for 18 hr according to the AOAC (2012), and results expressed as a percentage dry matter.

Lipids were extracted following the Bligh and Dyer method (1959) with modifications according to Iverson, Lang, and Couper (2001). The sample (5 g of fish mince) was homogenized in chloroform, methanol, and water mixture (1:1:0.8 v/v) at the speed of 226 *g* for 90 s using an Ultra Turrax homogenizer (T25 Homogenizer, Staufen, Germany). The homogenate was centrifuged at 1,595 *g* at 18°C for 10 min using a centrifuge (Sigma 4K15, Osterode am Harz, Germany) to obtain the extract (Chloroform phase). The lipid content was determined by gravimetry after evaporation of chloroform and expressed as percentage of dried fish sample.

#### 2.2.2 Primary and secondary lipid oxidation products

Peroxide values (PV) of the lipid extracts were determined according to the method of Shantha and Decker (1994) based on the formation of an iron−thiocyanate complex. The colored complex was measured by spectrophotometer (Shimadzu UV1800, Shimadzu Scientific Instruments, Columbia, MD) at 500 nm. The analysis was done in duplicate, and the results were expressed in milliequivalent peroxides/Kg oil (meq O_2_/Kg oil).

The volatile compounds from fish mince were collected using the dynamic headspace technique. The procedure was carried out using 1 g of fish mince in which 30 mg of internal standard, 4‐methyl‐1‐pentanol were added and mixed with 15 ml of distilled water. The volatiles were collected in Tenax GR tubes at 37°C by purging with nitrogen for 30 min at 150 ml/min. The tubes were flushed with nitrogen at 50 ml/min for 20 min to remove water. The trapped volatiles were desorbed from the Tenax tubes by heat (200°C) using an automatic thermal desorber (ATD‐400, PerkinElmer, Norwalk, CT), cryofocused on a cold trap (−30°C), released again at 220°C, and led to a GC an Agilent 5890IIA model (Palo Alto, CA, USA) equipped with a HP 5972 mass selective detector. Separation was done on a DB1701 column (30 m × ID 0.25 mm × 0.5 μm film thickness; J&W Scientific, Folsom, CA). The carrier gas used was helium at flow rate of 1.3 ml/min. The oven temperature was rising by 2.0°C/min from initial temperature of 45–80°C followed by an increase of 3.0°C/min to 150°C and finally increased by 12.0°C/min to 240°C. The individual compounds were identified by MS‐library searches and addition of the internal standard. Quantification was done through calibration curve made by adding the standard directly on the Tenax tubes as described by Nielsen, Debnath, and Jacobsen (2007). For the quantification, a stock solution of 19 volatiles was prepared and a calibration curve was conducted in a range from 0 to 1.2 mg/g. The analysis was carried out in triplicate.

#### 2.2.3 Free fatty acids and fatty acid profile

Free fatty acids (FFAs) content was determined by acidometric titration of the lipid extract using NaOH (0.1 M). The FFAs content was calculated as oleic acid according to the AOCS (2009), and results were reported as % oleic acid.

The fatty acid profile was determined as fatty acid methyl esters (FAMEs) according to the American Oil Chemists’ Society (AOCS) official method, Ce 1i‐07 (AOCS, 2009), with some modification as follows. Approximately 1 g of extract was weighed in a methylation glass tube and evaporated to dryness under a gentle stream of nitrogen. Thereafter, 100 µl of internal standard solution (2% w/v C23:0 in heptane), 200 µl of heptane including 0.01% w/v butylated hydroxytoluene (BHT) as antioxidant, 100 µl of toluene, and 1 ml of boron trifluoride in methanol (BF3‐MeOH) were added. Samples were mixed and methylated in the microwave oven (Microwave 3000 SOLV, Anton Paar) for 10 min at 100°C and power of 500 W and then cooled down for 5 min. Then, 1 ml of saturated salt water (NaCl) and 0.7 ml of heptane with BHT were added. After the separation of heptane, the upper phase of the sample (around 0.7 ml) was transferred into vials. Samples were analyzed by gas chromatography system (HP‐5890 A, Agilent Technologies, Santa Clara, CA, USA). Fatty acid methyl esters were separated and detected by the GC column Agilent DB‐wax (10 m × 100 µm × 0.1 µm), from Agilent Technologies (CA, USA). The carrier gas was helium with a flow rate of 0.38 ml/min and an inlet pressure of 51 psi. The oven temperature program for separation was from 160 to 200°C, then from 200 to 220°C, and from 220 to 240°C at 10.6°C/min. All analyses were done in duplicate. The result of each fatty acid was expressed as g fatty acid/100 g lipid.

#### 2.2.4 Antioxidant activity of clove and seaweed water extracts

##### Total phenolic content

The total phenolic compounds of the extracts were determined using Folin–Ciocalteu reagent by a procedure described by Farvin and Jacobsen (2013) in which gallic acid was used as a standard. The standard curve was prepared in distilled water at a concentration range of 0–125 µg/ml. The original extracts were diluted with water as necessary to fit within the standard curve. The absorbance was read at 725 nm using UV–vis spectrophotometer and results reported in µg gallic acid equivalent (µg GAE)/ml of clove and seaweed water extracts. All measurements were performed in duplicate.

##### Free radical scavenging ability

The free radical scavenging activities of clove and seaweed water extracts were measured by utilizing the stable radical, 1,1‐diphenyl‐2‐picrylhydrazil (DPPH) as described by Yang, Guo, & Yuan, 2008. The solutions of prepared extracts were diluted with water *(*1:1 v/v*)*. Diluted solutions (100 µl) were added to the microplate and mixed with 100 µl of 0.1 mM DPPH in ethanol (96%). The mixtures were shaken vigorously and maintained for 30 min at ambient temperature in the dark. The absorbance of mixtures and the control (100 µl DPPH solution + 100 µl BHT) was measured at 517 nm against a reagent blank by using a UV–vis spectrophotometer. The scavenging activity was calculated as inhibition percent by using the following equation:

$$\text{Inhibition}\left(\%\right)=\left(1-\frac{A_{\text{s}}-A_{0}}{A_{\text{b}}}\right)\times 100$$

where *A*_s_ is the absorbance of DPPH after reaction with antioxidant, *A*_0_ is the absorbance of antioxidant and ethanol (blank), and *A*_b_ is the absorbance of water and DPPH (blind).

##### Iron (Fe^2+^) chelating ability

The ferrous ion chelating activity of clove and seaweed extracts was measured as described by Farvin, Baron, Nielsen, and Jacobsen (2010) with 20 µl of 0.5 mM ferrous chloride and 20 µl of 2.5 mM ferrozin being mixed with 100 µl of clove and seaweed extracts. The mixture was allowed to equilibrate in the darkness at room temperature for 10 min before measuring the absorbance. The decrease in the absorbance at 562 nm of the iron (II)‐ferrozin complex was measured. EDTA was used as the positive control, and the ability of the extracts to chelate Fe^2^ *^+^* was calculated using the equation:

$${\text{Fe}}^{2+}\text{chelating activity}=\left(\frac{A_{\text{blank}}-\left(A_{\text{sample}}-A_{\text{blind}}\right)}{A_{\text{blank}}}\right)\times 100$$

where *A*_blank_ is the absorbance of blank (only iron chloride and Ferrozin), *A*_sample_ is the absorbance of sample, and *A*_blind _is the absorbance of blind (only antioxidant).

### 2.3 Statistical analysis

Data were analyzed using IBM SPSS (SPSS for Windows Version 20.0, 2013, IBM, Bethesda, MD, USA). Data were reported as mean ± standard deviation. Differences between means were determined using one‐way analysis of variance (one‐way ANOVA) with Tukey's HSD post hoc test, according to the equal variance of different groups. The correlations among variables were determined using a two‐tailed Pearson correlation coefficient. A *p*‐value <0.05 was considered statistically significant.

## 3 RESULTS AND DISCUSSION

### 3.1 Antioxidant activity of clove and seaweed water extracts

The clove and red seaweed (*Kappaphycusalvarezii)* water extracts analyzed in this study had total phenolic content levels in the range from 18.18 to 28.75 and 4.47 to 7.09 µgGAE/ml, respectively (Table 1). As expected in both cases, the 20 g/L extracts had significantly higher total phenolic content than that of 5 and 10 g/L. The total phenolic content in clove and seaweed extracts did not increase linearly with the amount of dry clove and seaweed extracted in 1 L of water. This suggests that longer time periods were needed for efficient extraction of phenolic compounds when larger amounts of dry clove and seaweed powder were used. Previous studies have shown that successful recovery of phenolic compounds from plant matrices by aqueous extraction depends on factors such as temperature, extraction time, and solvent to solid ratio (Çam & Aaby, 2010). The DPPH assay measures the ability of the extracts to donate hydrogen to the DPPH radical, resulting in bleaching of the DPPH solution. In our study, clove extracts had significantly higher (93%–95%) inhibition of DPPH than seaweed (29%–51%) inhibition. This could be due to higher phenolic content in clove than seaweed extracts although there was no linear relationship between total phenolic content and DPPH in clove extracts suggesting compounds other than phenolics (e.g., flavonoids) contributed to the antioxidant activity of clove extract. The DPPH decreased from 95.59% to 94.34% in clove extract and from 51.56% to 29.67% in seaweed extract when the amounts of clove and seaweed extracted in one liter of hot water were increased from 10 to 20 g.This could be due to decreases in extraction efficiency of clove and seaweed phenolics in boiling water at concentration above 10 g/L as reported in another study (Slavin, Dong, & Gewa, 2016). Elsewhere, clove water extract has been found to contain substantial amounts of phenolic compounds and powerful antioxidant activity in linoleic acid emulsion (Gülçin, Şat, Beydemir, Elmastaş, & Küfrevioǧlu, 2004).

**Table 1:** Antioxidant capacity of different doses of clove and seaweed water extracts

Extracts (g/L)	Total phenolic content (µgGAE/ml)	DPPH scavenging activity (% inhibition)	Fe^2+^ ion chelating activity (%)
CL 5	18.18^a^ ± 1.29	93.33^u^ ± 0.21	14.74^n ^± 4.60
CL 10	25.94^b^ ± 2.62	95.59^v^ ± 1.44	20.87^p ^± 5.98
CL 20	28.75^c^ ± 1.35	94.34^w^ ± 0.38	22.24^q ^± 4.08
SW 5	4.47^d^ ± 1.48	43.19^x^ ± 1.81	39.09^r^ ± 1.78
SW 10	6.82^e^ ± 2.64	51.56^y^ ± 1.04	38.39^s^ ± 3.93
SW 20	7.09^f^ ± 0.04	29.67^z^ ± 0.76	49.75^t^ ± 2.67

Note

Means marked with different letters in a column are statistically significant (*p* < 0.05).

CL: Clove; GAE: gallic acid; SW: seaweed; 5, 10, and 20: grams of seaweed or clove extracted.

The low DPPH radical scavenging activity of seaweed in the present study was in relative agreement with another study on antioxidant potential of red seaweed extract obtained using water as extraction solvent (Rao, Suresh, & Ganesan, 2008).

Seaweed extracts had significantly higher iron chelating activity than clove extracts (Table 1). The chelating activity increased (though not linearly) with the amount of clove extracted. The dependence of iron chelating capacity on concentration of clove water extracts and type of solvent used was reported by Gülçin et al. (2004). Essential oils of clove have been tested in omega‐6 and omega‐3 fatty acids enriched food supplements and found to have high radical scavenging activity, iron chelating properties, and higher hydrogen donating power than the standard antioxidants BHT and α‐tocopherol (Bag & Chattopadhyay, 2017).The high iron chelating capacity of seaweed extracts given their low total phenolic content suggests that other compounds such as seaweed polysaccharides extracted in boiling water may act as metal chelators.

### 3.2 Fat, free fatty acids, and dry matter content

The dry matter content of clove and seaweed was 86.40% and 80.30%, respectively. The mean dry matter content in sardines treated with seaweed was higher than in the control and clove pretreated sardines (Table 2). This could be due to differences in clarity of extracts from the two matrices after being gravity filtered. The seaweed extracts visually appeared to be more viscous compared to those from clove probably due to gelatinization of polysaccharides during hot water extraction and that could favor its adherence onto the surface of the product. Fat content in the samples ranged from 16.18% to 17.65%. This is in agreement with observations in our previous study in which we found that sardines dried on raised plat forms had 17.39% fat content (Chaula et al., 2019). In both clove‐ and seaweed‐treated samples, free fatty acids seemed to decrease with increasing concentration of the extracts suggesting that extracts limited lipolysis.

**Table 2:** Fat, free fatty acids, and dry matter content in sun‐dried sardines pretreated with clove and seaweed water extracts

Sample	Fat content (%)	Free fatty acids (%)	Dry matter (%)
Control	17.30^b^ ± 0.19	9.50^d^ ± 0.96	92.27^h^ ± 0.64
CL 5	17.65^b^ ± 0.13	14.84^d^ ± 2.06	91.63^h^ ± 0.92
CL 10	17.52^b^ ± 0.43	13.73^d^ ± 2.01	92.61^h^ ± 0.48
CL 20	16.28^c ^± 0.10	11.08^d^ ± 0.20	92.34^h^ ± 0.25
SW 5	16.18^c^ ± 0.25	10.75^f^ ± 2.54	93.70^i^ ± 0.14
SW 10	17.16^b^ ± 0.19	7.97^g^ ± 0.89	93.81^i^ ± 0.12
SW 20	17.19^b^ ± 0.16	7.58^g^ ± 1.05	93.92^i^ ± 0.13

Note

Values are expressed in mean ± standard deviation (*n* = 4). Means marked with different letters in a column are statistically significant (*p* < 0.05).

CL: clove; SW: seaweed; 5, 10, and 20: grams of seaweed and clove extracted.

### 3.3 Lipid peroxidation and volatile components

**Figure 1:**
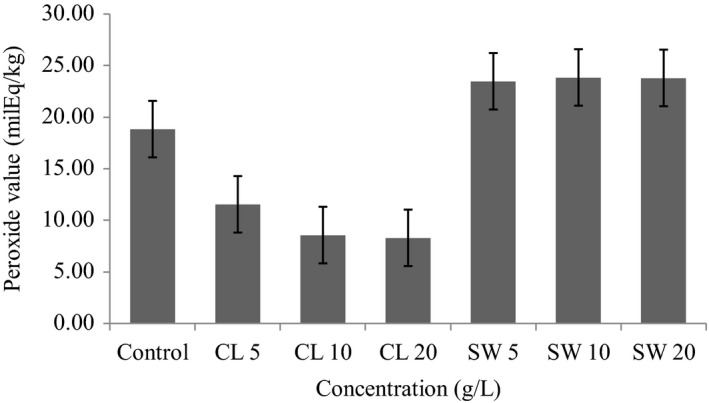
Peroxide value in sun‐dried sardines pretreated with different concentrations of clove and seaweed extracts immediately after drying. CL: clove; SW: seaweed; 5, 10, and 20: grams of seaweed and clove extracted in 1 L water

The peroxide value (PV) and the volatiles analyses were used to determine the primary and secondary lipid oxidation products in control and pretreated sardines after sun drying. From Figure 1, it can be seen that the control sample had PV of 18.83 mequiv.O_2_/Kg oil. Clove‐treated sardines had significantly lower peroxide values (ranging from 8.29 to 11.54 mequiv.O_2_/Kg oil) and concentrations of most of volatile compounds (Table 3) than the control. The PV values and the concentrations of volatile secondary oxidation products among clove‐treated samples decreased as the amount of clove extracted in 1 L of water increased. Soaking sardines in 5, 10, and 20 g/L clove water extracts for 40 min prior to sun drying significantly reduced peroxide values in dry sardine by 38.7%, 54.6%, and 56%, respectively, relative to the control. The pretreatments resulted into remarkable decrease in concentrations of individual volatile compounds, including 1‐penten‐3‐ol, 4‐heptanal and t, t‐2, 4‐heptadienal which are among the recognized decomposition products of EPA and DHA (Nielsen, Petersen, Meyer, Timm‐Heinrich, and Jacobsen, 2004). These observations indicate that lipid oxidation reactions were more pronounced in untreated sardines than in clove‐treated counterpart. Although there was no linear relationship between the amount of clove extracted in 1 L of water and the total phenolic compounds, higher clove extract concentrations had higher amounts of these compounds such that CL20 > CL10 > CL5. The reduced peroxide values and concentration of volatile compounds in clove‐treated samples given the high free radical scavenging capacity of clove extracts suggest that phenolic compounds in the extracts played an anti‐oxidative role during drying of sardines. Phenolic compounds are known to exert anti‐oxidative effect by different mechanisms such as scavenging of free radicals, singlet oxygen quenching, oxygen scavenging, metal chelation, and inhibition of oxidizing enzymes (Dudonné et al., 2009; Shobana & Akhilender, 2000). In literature, it has been shown that the use of the whole spices and herbs or their extracts with strong antioxidant activity (Gachkar et al., 2007) can control lipid oxidation in muscle food such as mullet fish, frozen chub mackerel, and smoked rainbow trout (Emir Çoban, Patir, & Yilmaz, 2014). When clove essential oils were used during refrigerated storage (at 2°C) of sliced smoked and vacuum packed rainbow trout (*Oncorhynchus mykiss*), a dose‐dependent reduction in peroxide values was observed (Emir Çoban & Patir, 2013).

**Table 3:** Concentration of volatile compounds (ng/g fish mince) in sun‐dried sardines pretreated with different concentrations of clove and seaweed extracts

Compound	Control	CL 5	CL 10	CL 20	SW 5	SW 10	SW 20
4‐heptenal	17.99^na^ ± 0.96	13.11^nb^ ± 2.19	14.51^nc^ ± 0.38	12.03^nd^ ± 1.33	23.26^ne^ ± 2.69	21.14^nf^ ± 1.42	23.19^ng^ ± 0.80
2‐heptenal	35.71^gb^ ± 14.05	8.21^gc^ ± 4.25	9.56^gd ^± 3.19	4.11^ge^ ± 1.65	42.23^gf^ ± 0.79	51.09^gh ^± 0.98	66.36^gi^ ± 8.19
2‐methyl furan	10.83^qn^ ± 1.43	0.91^qp^ ± 0.39	5.51^qq^ ± 1.07	ND	32.49^qr^ ± 5.88	23.77^qs^ ± 7.88	25.17^qu^ ± 8.84
t,t‐2,4‐heptadienal	5.66^ww^ ± 0.47	3.39^wx^ ± 0.67	2.85^wy^ ± 1.09	2.16^wz^ ± 1.89	8.91^ab^ ± 2.29	8.21^ab^ ± 0.91	10.18^ac^ ± 1.31
2‐pentenal	196.49^aa^ ± 9.27	84.07^bb^ ± 11.43	80.84^cc^ ± 7.82	44.75^dd^ ± 4.27	273.52^ee^ ± 96.24	261.44^ff^ ± 27.29	328.79^gg^ ± 51.75
2‐heptanon	130.21^ab^ ± 5.81	73.76^ac^ ± 9.73	84.33^ad^ ± 5.32	45.36^ae^ ± 2.76	169.44^af^ ± 30.89	210.49^ag^ ± 15.45	130.58^ah^ ± 16.99
1‐penten‐3‐ol	276.02^bc^ ± 1.45	196.33^bd^ ± 24.49	182.25^be^ ± 24.59	125.77^bf^ ± 10.08	370.25^bg^ ± 44.49	321.40^bh^ ± 32.53	374.85^bj ^± 62.74
Benzaldehyde	370.66^pa^ ± 34.34	286.79^pb^ ± 44.23	255.91^pc^ ± 27.07	242.21^pd ^± 10.26	471.26^pe^ ± 44.09	448.07^pf^ ± 72.69	536.78^pg^ ± 87.17
Butanal	876.53^rb^ ± 56.46	405.19^rc^ ± 83.31	383.28^rd^ ± 43.97	208.17^re^ ± 4.71	1,797.59^rf^ ± 167.27	1,494.39^rg^ ± 286.11	1,659.53^rh ^± 155.68
2‐methylbutanal	946.93^wa^ ± 50.38	254.16^wb ^± 40.22	236.92^wc^ ± 18.91	178.05^wd^ ± 5.34	984.94^we ^± 117.58	1,042.15^wf^ ± 156.97	985.58^wg^ ± 292.36
t‐2‐penten‐1‐ol	2,175.65^sb^ ± 83.32	844.77^sc^ ± 83.35	825.76^sd^ ± 45.55	439.22^se^ ± 1.90	3,137.69^sf ^± 297.01	2,786.05^sg^ ± 116.54	2,911.91^sh^ ± 58.96
Hexanal	2,628.79^a ^± 205.95	1,935.43^b ^± 196.86	1,900.45^c ^± 121.14	965.62^d^ ± 24.08	5,477.66^e^ ± 598.93	6,026.30^f^ ± 359.96	5,248.99^g^ ± 267.63
Pentanal	1,579.58^h^ ± 18.55	1,224.85^i ^± 149.74	1,123.31^j^ ± 90.05	783.12^l^ ± 38.36	2,178.92^m ^± 166.30	1,921.86^n^ ± 136.39	2,112.39^p^ ± 367.39
1‐octene‐3‐ol	983.34^r ^± 67.94	389.07^s^ ± 69.13	382.38^t^ ± 33.81	247.12^u^ ± 1.79	1,201.52^v ^± 274.31	1,184.45^w^ ± 116.36	1,293.13^x^ ± 205.62
3‐methylbutanal	2,111.63^é^ ± 95.19	1,695.66^y^ ± 248.54	1,591.88^z ^± 114.12	1,208.99^é ^± 32.97	2,273.81^o^ ± 220.83	2,317.76^zp^ ± 300.57	2,262.23^zq^ ± 596.96
Heptanal	1,104.58^xa^ ± 36.39	398.86^xb^ ± 58.20	442.16^xc ^± 27.91	268.52^xd ^± 13.53	1,575.01^xe ^± 216.46	1,371.07^xf^ ± 47.85	1,487.51^xg^ ± 135.92

Note

Values are expressed in mean ± standard deviation (*n* = 3). Means marked with different letters in a row are statistically significant (*p* < 0.05).

CL: clove; ND: not detected; SW: seaweed; 5, 10, and 20: grams of seaweed and clove extracted in 1 L of boiling water.

Seaweed‐treated samples had significantly higher PV values and concentrations of volatile compounds relative to the control. There was no significant difference in PV values among samples treated with 5, 10, and 20 g/L doses of seaweed water extracts. Seaweed extracts had a higher metal chelating activity than the clove extracts but a lower DPPH scavenging activity. Thus, higher PV values and concentrations of volatiles in seaweed pretreated sardines suggest that metal chelating is a less important anti‐oxidative mechanism in sun‐dried sardines than radical scavenging or that the metal chelating compounds extracted from seaweed were less accessible than in the clove. Another possibility is that the seaweed polysaccharides extracted had high reducing power which could have reduced Fe^3+^ to Fe^2+^ there by promoting oxidation. Seaweeds are known for their capacity to concentrate calcium and magnesium salts. When milled and extracted with water at 90°C, *Kappaphycus alvarezii* was found to contain Na^+^ (20.2%), K^+^ (1.7%), Ca^2+^ (59.2%), and Mg^2+^ (18.2%) (José, Marina, & Alberto, 2004). Fish muscles are prone to lipid oxidation catalyzed by presence of metal ions. The fact that seaweed water extract contains major amounts (77.9%) of divalent (Ca^2+ ^and Mg^2+^) cations could have contributed to increased PV values and concentrations of volatile compounds in seaweed pretreated sardines.

Different brown and red seaweed species from Danish coast were screened for antioxidant activity and phenolic compounds and tested in real food systems. One red seaweed (*P. fucoides*) was found to have high phenolic content and good antioxidant activity in all in vitro assays, was effective in preventing oxidation of fish oil in all food systems tested, and was effective even at a temperature of 60°C and high oxygen pressure (Farvin and Jacobsen, unpublished).

However, it should be noted that in the fish matrix there can be complex molecular interactions. For example, in the presence of secondary lipid oxidation products (aldehydes) oxidative deamination of α‐amino acids in proteins (Strecker degradation) can occur resulting in formation of Strecker aldehydes (Zamora & Hidalgo, 2011; Lu, Nielsen, Baron, & Jacobsen, 2017). In this study, we could quantify the volatiles 3‐methylbutanal and 2‐methylbutanal as well as benzaldehyde which have been reported to be strecker aldehydes in sea foods containing primary amine groups (Lu, Bruheim, Haugsgjerd, & Jacobsen, 2014; Thomsen et al., 2013). Nevertheless, more information on metal chelating activity and reducing power of polysaccharides in hot water extracts of red seaweed as well as the possible presence of volatiles in the extract is needed.

### 3.4 Fatty acid profiles

A total of 28 fatty acids were identified and quantified in the lipid extracts of both control and pretreated sardines (Table 4). The SFAs were relatively fewer (6) compared to unsaturated (22). Palmitic acid (C16:0) and stearic acid (C18:0) were found to constitute 21.1%–26.2% and 7.9%–9.8% of the total lipid content, respectively. Thirteen of the 22 unsaturated fatty acids were PUFAs, and nine were MUFAs. Among the thirteen PUFAs, the omega‐3 fatty acids were relatively more abundant (6), followed by omega‐6 (4). There was no significant difference in total SFAs and MUFAs between the control and samples treated with seaweed extracts. However, sardines pretreated with clove extracts registered significantly lower total SFAs and MUFAs than the untreated. The total PUFAs ranged from 29.8% to 31.9% and 21.4% to 23.3% in clove and seaweed pretreated sardines, respectively. Clove pretreatment resulted in significantly higher retention of total PUFAs in dried sardines. There was no significant difference in total PUFAs between the control and the seaweed pretreated samples. The polyene index (PI) value is a ratio of PUFAs amount to that of the relatively stable C16:0 and is used here to indicate and compare damage to PUFAs during sun drying of untreated (control), clove and seaweed pretreated sardines. Higher PI values in clove pretreated sardines showed improved retention of long‐chain polyunsaturated fatty acids. Correspondingly, these samples had significantly higher content of the three nutritionally valued omega‐3 PUFAs, DHA (12.7%–13.4%), DPA (2.0%–2.1%), and EPA (5.4%–5.7%) than the control and seaweed pretreated samples, which had 7.8%–8.7%, 1.2%–1.4%, and 3.3%–3.6% of DHA, DPA, and EPA, respectively. Lower proportions of DHA, DPA, EPA, and lesser unsaturated fatty acids in lipid fractions of untreated and seaweed‐treated sardine are evidences of lipid oxidation therein. There was no significant difference in total amount of omega‐6 fatty acids between pretreated and control samples.

**Table 4:** Fatty acid profiles (% fatty acid of total fatty acids) of sun‐dried sardine pretreated with different concentrations of clove and seaweed water extracts

Fatty acid	Control	CL 5	CL 10	CL 20	SW 5	SW 10	SW 20
14:0	4.09 ± 0.06	3.72 ± 0.07	3.69 ± 0.04	3.54 ± 0.01	4.06 ± 0.03	4.06 ± 0.06	4.37 ± 0.07
15:0	0.89 ± 0.04	0.71 ± 0.01	0.68 ± 0.03	0.72 ± 0.01	0.87 ± 0.01	0.88 ± 0.01	0.90 ± 0.02
16:0	24.88 ± 0.42	21.61 ± 0.26	21.05 ± 0.75	21.29 ± 0.01	25.13 ± 0.02	24.93 ± 0.11	25.68 ± 0.33
17:0	0.59 ± 0.03	0.46 ± 0.01	0.43412613	0.46 ± 0.01	0.63 ± 0.05	0.62 ± 0.05	0.58 ± 0.06
18:0	9.25 ± 0.12	8.13 ± 0.07	7.95 ± 0.27	8.08 ± 0.05	9.29 ± 0.05	9.18 ± 0.03	9.27 ± 0.14
24:0	0.17 ± 0.02	0.14 ± 0.02	0.26 ± 0.04	0.20 ± 0.01	0.15 ± 0.02	0.11 ± 0.08	0.16 ± 0.01
Total SFAs	39.86^a^ ± 9.57	34.76^b^ ± 8.32	34.08^c^ ± 8.08	34.30^c^ ± 8.19	40.15^a^ ± 9.66	39.75^a^ ± 9.59	40.07^a^ ± 10.07
14:1	0.06 ± 0.01	0.12 ± 0.04	0.08 ± 0.01	ND	0.39 ± 0.04	0.11 ± 0.01	0.12 ± 0.01
16:1 (n‐7)	9.95 ± 0.16	8.91 ± 0.11	8.51 ± 0.13	4.47 ± 0.62	9.79 ± 0.08	10.04 ± 0.07	10.28 ± 0.16
17:1	ND	0.23 ± 0.03	ND	ND	ND	ND	ND
18:1 (n‐9)	3.63 ± 0.03	3.11 ± 0.02	3.26 ± 0.22	3.20 ± 0.03	3.66 ± 0.06	3.73 ± 0.04	3.72 ± 0.10
18:1 (n‐7)	0.43 ± 0.05	0.32 ± 0.01	0.39 ± 0.03	0.34 ± 0.08	0.43 ± 0.03	0.48 ± 0.05	0.44 ± 0.05
20:1 (n‐9)	0.45 ± 0.01	0.26 ± 0.02	0.30 ± 0.04	0.29 ± 0.01	0.44 ± 0.06	0.46 ± 0.07	0.48 ± 0.04
20:1 (n‐7)	0.33 ± 0.04	0.29 ± 0.01	0.27 ± 0.01	0.28 ± 0.01	0.36 ± 0.08	0.36 ± 0.01	0.37 ± 0.02
22:1 (n‐11)	0.38 ± 0.02	0.34 ± 0.01	0.39 ± 0.04	0.38 ± 0.04	0.36 ± 0.02	0.38 ± 0.02	0.37 ± 0.02
24:1 (n‐9)	0.42 ± 0.03	0.37 ± 0.05	0.49 ± 0.07	0.21 ± 0.02	0.39 ± 0.02	0.39 ± 0.01	0.41 ± 0.02
Total MUFAs	15.66^f^ ± 3.43	13.94^g^ ± 2.92	13.70^g^ ± 2.81	9.18^h^ ± 1.63	15.84^f^ ± 3.21	15.95^f^ ± 3.31	16.19^f^ ± 3.38
16:2 (n‐4)	0.51 ± 0.01	0.64 ± 0.02	0.60 ± 0.02	0.61 ± 0.02	0.49 ± 0.09	0.51 ± 0.01	0.51 ± 0.05
16:3 (n‐4)	2.52 ± 0.02	2.24 ± 0.06	2.23 ± 0.08	2.26 ± 0.02	2.54 ± 0.03	2.54 ± 0.02	2.55 ± 0.03
18:3 (n‐4)	2.84 ± 0.08	1.81 ± 0.21	3.52 ± 0.11	3.46 ± 0.04	2.73 ± 0.13	2.83 ± 0.16	2.50 ± 0.09
Total (n‐4)	5.87^k^ ± 1.27	4.69^l^ ± 0.83	6.35^m^ ± 1.46	6.33^m^ ± 1.43	5.76^n^ ± 1.24	5.88^n^ ± 1.27	5.56^n^ ± 1.16
18:3 (n‐3)	0.46 ± 0.14	0.45 ± 0.01	0.43 ± 0.03	0.39 ± 0.05	0.37 ± 0.10	0.45 ± 0.16	0.38 ± 0.18
18:4 (n‐3)	ND	0.05 ± 0.01	0.05 ± 0.01	ND	0.06 ± 0.01	ND	ND
20:3 (n‐3)	0.43 ± 0.04	0.54 ± 0.01	0.54 ± 0.01	0.55 ± 0.02	0.41 ± 0.01	0.42 ± 0.01	0.38 ± 0.01
20:5 (n‐3)	3.62 ± 0.11	5.69 ± 0.29	5.44 ± 0.01	5.71 ± 0.03	3.52 ± 0.04	3.64 ± 0.06	3.33 ± 0.05
22:5 (n‐3)	1.37 ± 0.03	2.02 ± 0.04	2.10 ± 0.03	2.09 ± 0.01	1.32 ± 0.01	1.37 ± 0.01	1.21 ± 0.04
22:6 (n‐3)	8.71 ± 0.18	12.93 ± 0.58	12.68 ± 0.06	13.38 ± 0.02	8.51 ± 0.03	8.43 ± 0.01	7.82 ± 0.05
Total (n‐3)	14.59^p^ ± 3.49	21.68^q^ ± 5.02	21.24^q^ ± 4.90	22.15^r^ ± 5.19	14.19^s^ ± 3.26	14.30^s^ ± 3.24	13.14^t^ ± 3.01
18:2 (n‐6)	0.16 ± 0.06	0.12 ± 0.01	0.17 ± 0.04	0.13 ± 0.01	0.69 ± 0.07	0.17 ± 0.06	0.17 ± 0.01
18:3 (n‐6)	0.38 ± 0.08	0.31 ± 0.01	0.36 ± 0.05	0.29 ± 0.01	0.41 ± 0.01	0.38 ± 0.11	0.62 ± 0.07
20:2 (n‐6)	0.23 ± 0.01	0.23 ± 0.01	0.24 ± 0.01	0.23 ± 0.02	0.20 ± 0.01	0.22 ± 0.03	0.20 ± 0.01
20:4 (n‐6)	2.11 ± 0.05	2.78 ± 0.21	2.64 ± 0.05	2.81 ± 0.01	2.03 ± 0.03	2.06 ± 0.06	2.88 ± 0.04
Total (n‐6)	2.87^v^ ± 0.93	3.44^v^ ± 1.28	3.41^v^ ± 1.19	3.46^v^ ± 1.29	3.34^v^ ± 0.82	2.84^v^ ± 0.90	2.82^v^ ± 0.82
Total PUFAs	23.34^w^ ± 2.44	29.81^y^ ± 3.56	31.00^y^ ± 3.49	31.94^y^ ± 3.69	23.29^w^ ± 2.31	23.01^w^ ± 2.33	22.93^w^ ± 2.15
PI	0.94	1.38	1.47	1.50	0.93	0.92	0.82

Note

Values are expressed in mean ± standard deviation (*n* = 4). Total PUFAs = total (n‐4) + total (n‐3) + total (n‐6). Means marked with different letters in the same row are statistically significant (*p* < 0.05).

MUFA: monounsaturated fatty acids; ND: not detected; PI: polyene index; PUFA: polyunsaturated fatty acids; SFA: saturated fatty acids.

^a^Fish lipid was extracted from whole fish.

Clove has been reported to have high phenolic content and antioxidant components with high thermal stability (Shan, Cai, Sun, & Corke, 2005; Shobana & Akhilender, 2000). The use of spices like clove as natural antioxidant to protect lipids in meat and fish oil has been demonstrated (Falowo, Fayemi, & Muchenje, 2014; Shah, Bosco, & Mir, 2014). Improved retention of long‐chain polyunsaturated fats and preservation of omega‐3 fatty acids in oven dried sardine (*R. argentae*) pretreated with clove water extracts has also been shown (Slavin et al., 2016). Clove oil seems also a powerful antioxidant in the linoleic acid system with its inhibition potential being concentration‐dependent (Gülçin, Elmastaş, & Hassan, 2012). Water extracts of clove are also reported to have as strong peroxidation inhibitory effect as ethanol extract in linoleic acid emulsion (Gülçin et al., 2004). The antioxidant activity of clove extracts may be attributed to strong hydrogen donating ability, metal chelating ability, and effectiveness as free radicals scavenger. In addition, clove extracts had higher (×4) total phenolic compounds than the seaweed extracts, which appears to be responsible for the antioxidant activity of clove extracts. However, the major phenolic compounds in clove are phenolic acids such as flavonol glucosides, phenolic volatile oils, and tannins, recovery of which is highly dependent on extraction conditions, differences in solvent, and extraction method (Dudonné et al., 2009; Shan et al., 2005; Wu et al., 2004).

## 4 CONCLUSIONS

The present study evaluated the efficacy of clove and seaweed water extracts to retard lipid oxidation in sun‐dried sardines. It was found feasible to pretreat whole, omega‐3‐rich small sardines with natural antioxidants to prevent lipid oxidation during sun drying. Both clove and seaweed water extracts had significant effect on chemical indicators of lipid oxidation in sun‐dried sardines. Pretreatment of sardine with clove water extracts resulted in improved retention of nutritionally valued long‐chain polyunsaturated fatty acids, particularly the omega‐3 fatty acids DHA, EPA, and DPA. However, the success of these pretreatments to impede lipid oxidation may partly be attributed to small size and weight of sardine fish. These findings would be of interest during incorporation of sardines into other food product formulation at industrial scale for product diversification. Nevertheless, for extended storage of sardines, further investigation on lipid oxidation of clove pretreated dry product may be needed. Such studies should also include further characterization of the clove extract to understand the mechanisms behind its anti‐oxidative effect and possible alterations in products’ sensory acceptability. Seaweed water extracts pretreatment resulted in low retention of polyunsaturated fatty acids in sun‐dried sardines. The pro‐oxidant activity of red seaweed water extracts needs to be further investigated by characterizing pigments, trace metals, and other polysaccharides likely to be responsible for this observation.

## CONFLICT OF INTEREST

The authors declare that there are no conflicts of interest.

## ETHICAL STATEMENT

This study did not involve human or animal subjects, and therefore, no human or animal testing was necessary.
